# Visual Function Characteristics in *STXBP1* Epileptic Encephalopathy Patients

**DOI:** 10.3390/jcm14196840

**Published:** 2025-09-26

**Authors:** Agnieszka Rosa, Dominika Nowakowska, Piotr Rosa, Justyna Simiera, Andrzej Gliniany, Michał Zawadka, Krzysztof Szczałuba, Lukasz Przyslo, Krystyna Szymańska, Piotr Loba, Maciej Gawęcki, Dorota Pojda-Wilczek

**Affiliations:** 1 Department of Pediatric Neurology, Pediatrics and Rare Disorders, Medical University of Warsaw, 02-091 Warsaw, Poland; neurolog.zawadka@gmail.com (M.Z.); krzysztof.szczaluba@wum.edu.pl (K.S.); krystyna.szymanska@uckwum.pl (K.S.); 2 Center of Excellence for Rare and Undiagnosed Disorders, Medical University of Warsaw, 02-091 Warsaw, Poland; piotr.rosa@orticus.com; 3 Department of General and Pediatric Ophthalmology, Medical University of Lublin, 20-059 Lublin, Poland; dominika.nowakowska85@gmail.com; 4 Department of Binocular Vision Pathophysiology and Strabismus, Medical University of Lodz, University Barlicki Hospital No. 1, 90-153 Lodz, Poland; justynaolcz@gmail.com (J.S.); ploba@onet.pl (P.L.); 5 Department of Developmental Neurology and Epileptology, Research Institute of Polish Mother’s Memorial Hospital, 93-338 Lodz, Poland; angrzej.gliniany@iczmp.edu.pl (A.G.); l.przyslo@gmail.com (L.P.); 6 Dobry Wzrok Ophthalmological Center, 80-392 Gdansk, Poland; maciej@gawecki.com; 7 Department of Ophthalmology, Faculty of Medical Sciences in Katowice, Medical University of Silesia, 40-055 Katowice, Poland; pojda-wilczek@wp.pl

**Keywords:** STXBP1 synaptopathy, epileptic encephalopathy, visual phenotype, personalized medicine

## Abstract

**Background**: The goal of the study was to describe the visual function characteristics of children with developmental epileptic encephalopathy resulting from mutations in the *STXBP1* gene. **Methods**: The study included 26 consecutive patients from the Polish STXBP1 population (11 male and 16 female; mean age: 7 years and 4 months; SD 4.03; range: 2–16 years) evaluated at a single center for strabismus and binocular vision. Data were obtained from medical records, including ophthalmological, neurological, and genetic information, as well as orthoptic and ophthalmological examinations performed in the clinic. **Results**: No major eye disorders were identified during the ophthalmological evaluation. The average prevalence of hyperopia was 76.9% (mean for OD, OS), with hyperopia above 4.25 D occurring in 17.3% (*n* = 4) of participants. Astigmatism was present in 96.2% of patients, with values ≥ 2.75 D in 27% (*n* = 7) of the group. The mean disc–foveal angle across all subjects was 7.23° ± 6.85° (range: −10.34° to 19.77°). Convergence was absent in 53.8% (*n* = 14) of patients. Mean accommodation responses equal to or higher than +1.0 D in any eye were noted in 90.5% of subjects. The mean accommodative/convergence (AC/A) ratio was 1.16 (SD 1.05; range: 0–3.3). Fusion was diagnosed using the 20 base-out prism test in 77% (*n* = 20) of patients, of which 85% (*n* = 17) had a positive response. **Conclusions**: This is the first study to comprehensively assess visual function in children with STXBP1 synaptopathy. Binocular vision development in individuals with STXBP1 differs from that of the general population. Considering the high prevalence of refractive errors, deficits in accommodation, and a low AC/A ratio, early visual diagnostics and the use of corrective eyewear are recommended in these patients.

## 1. Introduction

Synaptopathies are a group of epileptic or non-epileptic encephalopathies of various etiologies, with impaired synapse function as a common feature [1]. The function of synapses is dependent on proteins regulating the fusion of synaptic vesicles (and thus the release of neurotransmitters), recycling of vesicles, and expression of membrane receptors [2,3].

*STXBP1*-related developmental and epileptic encephalopathy (DEE4, *STXBP1* Epileptic Encephalopathy, OMIM #612164, Orpha: 599373) is a well-recognized synaptopathy resulting from mutations in the *STXBP1* gene [4,5]. The *STXBP1* protein (syntaxin-binding protein 1, Munc18-1) participates in the docking and fusion of synaptic vesicles. When the STXBP1 protein is impaired, exocytosis is disturbed, resulting in an imbalance between excitatory and inhibitory systems, leading to a decrease in the number of synapses, their strength, and plasticity [6].

Mutations in the *STXBP1* gene are associated with a wide spectrum of neurodevelopmental features, including developmental and speech delay, intellectual disability, hypotonia, various movement disorders (tremor, ataxia, bruxism, and dyskinesia), and behavioral abnormalities [7,8]. Epilepsy is diagnosed in approximately 95% of patients [4,5,7,8].

Visual abnormalities are common clinical signs in other synaptopathies, including developmental epileptic encephalopathies. In *DLG4*-synaptopathy, symptoms range from strabismus, hyperopia/myopia, and nystagmus to abnormal vision processing, including blindness [9].

The *STXBP1* protein is putatively involved in vision maintenance. Along with syntaxin 3, it is likely involved in retinal function, and a deficiency in these proteins results in progressive atrophic changes and a weakened response to light [10]. It is hypothesized that -/dysfunction may be related to the pathogenesis of congenital nystagmus [11].

Our trial is the first comprehensive analysis of eyesight function and visual abnormalities in *STXBP1* epileptic encephalopathy patients based on clinical, orthoptic, and ophthalmological assessment. We also used optical coherent tomography and cyclorotation, as measured through the size of the disc–foveal angle (DFA), which allowed us to characterize the unique visual phenotype of *STXBP1* synaptopathy.

## 2. Methods

### 2.1. Recruitment and Inclusion Criteria

This study included 26 consecutive patients diagnosed with STXBP1 epileptic encephalopathy based on genetic testing results. All patients were members of the STXBP1 Polska Association and directed for ophthalmological and orthoptic assessment by that institution. All examinations were conducted in April 2025.

### 2.2. Data Collection

The study was conducted at the Orticus Center for Strabismus and Binocular Vision Treatment (Grodzisk Mazowiecki, Poland) by an ophthalmologist and an orthoptist. All participants underwent both ophthalmological and orthoptic examinations, and their medical history was collected. Caregivers completed a questionnaire addressing prenatal, perinatal, and early developmental concerns that, in some cases, led to further diagnostic procedures.

### 2.3. Ophthalmological Examination

The ophthalmological assessment included a slit-lamp examination of the anterior segment, including eyelid positioning, and fundoscopy after mydriasis. Direct ophthalmoscopy and optical coherence tomography (OCT) of the macula and optic nerve head were performed using REVO FC Angio-OCT (Optopol, Zawiercie, Poland). Additionally, color and red-free fundus photographs were taken.

Objective cyclorotation was assessed by measuring the DFA. Digital fundus photographs of both eyes were captured under binocular viewing conditions while the subject fixated on an internal target. DFA values were calculated using Cyclocheck^®^ software (http://www.cyclocheck.com/, accessed on 12 July 2025) [12].

Refractive errors were evaluated before and after cycloplegia. Tropicamide 1% was used to paralyze accommodation, with three doses applied at 5 min intervals, because of the presence of epilepsy in the participants. Refractive measurements were taken 30 min after the final dose using a portable autorefractometer (Handy Ref-K, NIDEK Co., Ltd., Gamagori, Japan).

### 2.4. Orthoptic Examination

Orthoptic assessments were standardized and conducted by the same specialist using consistent equipment. Intellectual disabilities present in many patients required predominantly objective methods of assessment, as well as the exclusion of distance tests. Various fixation objects and illuminated toys were used to maintain patients’ engagement. Visual acuity testing with the Lea Test proved unreliable due to cognitive difficulties, so the Preferential Looking Test (Lea Test) was used for each eye at 50 cm.

The orthoptic examination included the following tests: (1) Eye movement evaluation in nine gaze directions. (2) Cover tests for near fixation using the standard prismatic cover test (PCT) and PCT with an additional +3.00 D lens. The difference in the strabismus angle was used to calculate the accommodative/convergence (AC/A) ratio using the gradient method. (3) Near point of convergence (NPC) tests, repeated three times for reliability, with measurements taken using a ruler based at the nose. (4) Fusion tests with a 20Δ base-out prism, followed by fusional range assessments using a prism bar, where fusional disruption was noted by observable fixation shifts rather than verbal feedback. (5) Dynamic retinoscopy mean accommodation response tests were performed monocularly at a working distance of 45–50 cm, using small, colorful pictures placed around the retinoscope’s light source to maintain the child’s attention. Two measurements were taken for each eye, and the average value was recorded. The lag or lead of accommodation was noted based on the movement of the reflex and the lens power required to neutralize it momentarily.

## 3. Statistical Analysis

Descriptive statistics were used according to the type of variable. Categorical variables are presented as frequencies (%), while continuous variables are reported as the mean (M), range (Min–Max), median (Me), and standard deviation (SD). The Shapiro–Wilk test was used to assess normality, with significance levels set at *p* < 0.05, *p* < 0.01, and *p* < 0.001. Relationships between age and visual parameters were analyzed using an analysis of variance (ANOVA) to compare age groups [*p* < 0.05, *p* < 0.01, and *p* < 0.001], and linear regression was used to evaluate trends (R^2^ and coefficient significance). To analyze both linear and monotonic relationships between age and refractive errors, the Spearman correlation test, the Pearson test, and the Kruskal–Wallis test were used. Data analysis was conducted using IBM SPSS Statistics, Version 29.0 (IBM Corp., Armonk, NY, USA) and Python, Version 3.11 (Python Software Fundation, Wilmington, DE, USA).

## 4. Results

### 4.1. Demographics

The study cohort included 26 participants aged 2–16 years (M = 7.4, SD = 4.03), with 10 males (M = 8.2, range: 2–16 years) and 16 females (M = 6.86, range: 1.6–16 years). The median age was 6.5 years. Children under 4 years comprised 19% (*n* = 5) of the cohort, 65.5% (*n* = 17) were aged 4–10 years, and 15.5% (*n* = 4) were aged over 10 years.

### 4.2. Pregnancy and Perinatal History

Caregiver-reported data indicated uneventful pregnancies in 88.5% (*n* = 23), with 80.8% (*n* = 21) of mothers having no significant health conditions. Hypothyroidism occurred in 19% (*n* = 5) and gestational diabetes in 7.7% (*n* = 2) of mothers. Delivery was normal in 84.7% (*n* = 22), with prolonged labor or low oxygen saturation in 11.5% (*n* = 3). Grade 3 brain hemorrhage was reported in one case (3.85%). The mean gestational age was 39.04 weeks (SD = 1.19), and most newborns scored 10 points on the Apgar scale (88.5%, *n* = 23; M = 9.85, SD = 0.46, range: 8–10 points).

### 4.3. Initial Symptoms and Diagnosis

The mean age at diagnosis was 4.3 years (range: 1 month to 12 years). Early symptoms included motor delays (96.2%, *n* = 25), seizures (92.4%, *n* = 24), hypotonia (88.6%, *n* = 23), cognitive delays (84.7%, *n* = 22), and impaired visual responses (61.6%, *n* = 16). Internal organ diseases, such as eosinophilic esophagitis and kidney failure, were diagnosed in 7.7% (*n* = 2) of patients. Multiple symptoms (≥4) co-occurred in 88.6% (*n* = 23) of patients. At the time of this study, seizures were present in 88.5% (*n* = 23) of patients.

**Figure 1:**
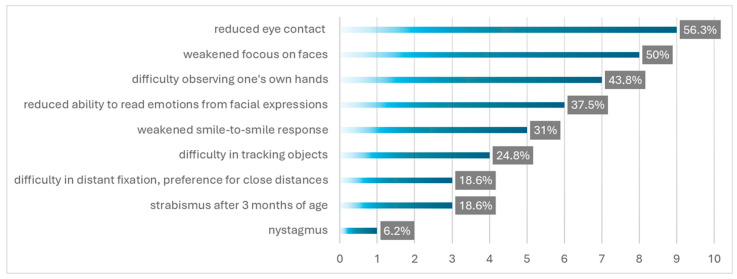
Frequency of alarming visual symptoms during the first months of life.

Caregivers reported visual concerns, including reduced eye contact (56.3%, *n* = 9), difficulty focusing on faces (50%, *n* = 8), problems observing hands (43.8%, *n* = 7), difficulty recognizing facial expressions (37.5%, *n* = 6), and a weakened smile-to-smile response (31%, *n* = 5; Figure 1).

### 4.4. Ophthalmological Findings

The anterior and posterior eye segments were evaluated in all patients. The anterior segment was normal in all cases. In the majority of patients, examination of the posterior segment revealed normal retinal vasculature and optic nerve discs with no signs of edema or atrophy. In three cases (11.6%), optic nerve protrusion and the absence of physiological cupping were noted, but no optic disc drusen were confirmed through additional OCT or B-scan ultrasonography.

Fundus photographs were successfully obtained from 88.6% (*n* = 23) of patients. Fundus photographs of 46 eyes were analyzed. In all but two eyes, the DFA was calculated. In one case, fundus imaging was not performed due to the child’s low level of cooperation, which prevented acquisition of adequate photographs. The mean DFA value in the whole study group (regardless of the site) was 7.23° ± 6.85° (median value 6.87°; range: −10.34° to 19.77°). The mean value of the DFA in the whole study group was 6.52° ± 7.11° (range: −10.34° to 19.12°) in the right eye and 7.59° ± 6.67° (range: −4.23° to 19.77°) in the left eye. OCT was performed in 61.6% (*n* = 16) of cases. The morphology of the macula and fovea was normal in all patients. One patient exhibited manifest latent nystagmus.

### 4.5. Refractive Errors

In the examined group, the mean prevalence of hyperopia in both eyes was 76.9%. The highest occurrence was within the range of 0.0 to 2.50 D (32.7%), followed by 26.9% of participants with hyperopia ranging from 2.75 to 4.0 D, and 17.3% with values exceeding 4.25 D (Table 1).

Myopia was identified in two participants (7.7%), whereas astigmatism was present in 96.2% (*n* = 25) of participants. Astigmatism is expressed as negative values; 48% (*n* = 13) of participants had astigmatism in the range 0.0–1.0 D, 21.2% in the range 1.25–2.50 D, and 27% had values exceeding 2.75 D (Table 2).

Based on medical history, an ophthalmological examination had previously been conducted in 73.2% (*n* = 19) of participants. Spectacle correction was used by 34.7% (*n* = 9) of patients. Following the examinations described here, spectacle correction was prescribed for 77% (*n* = 20) of participants.

### 4.6. Orthoptic Parameters

Eye movement in nine directions of gaze was normal in all participants, excluding extraocular muscle abnormalities. Strabismus was noted in 50% (*n* = 13), with 38.5% (*n* = 10) of patients presenting exophoria (PCT ≥ 10 PD range: 10–18 PD). Among the remaining patients, 3.85% (*n* = 1) had esophoria, 3.85% (*n* = 1) had non-accommodative esotropia, and one patient had exotropia.

Visual responses were assessed in 38.5% (*n* = 10) of participants using the Preferential Looking Test, with a mean OD/OS value of 3.45 (SD = 2.03; range: 0.50–8.0). Convergence was absent in 53.8% (*n* = 14), significantly reduced (NPC ≥ 11 cm) in 11.55% (*n* = 3), and normal (NPC ≤ 10 cm) in the remaining patients.

Fusion (assessed using the 20 base-out prism test) was evaluated in 77% (*n* = 20) of participants, with a positive response in 85% (*n* = 17) and an absence of the fusion reflex in 15% (*n* = 3) of patients. Similarly, fusion ranges were assessed in 20 participants, with a mean value of 28 PD (SD = 6.26) for convergence fusion and 17.12 PD (SD = 5.15) for divergence fusion.

Dynamic retinoscopy was performed in all participants. The mean value in the right eye (OD) was 2.63 (SD = 1.24; range: +0.75 to +6.00), while in the left eye (OS), it was 2.68 (SD = 1.31; range: +0.75 to +6.50). An accommodative response of ≥+1.0 D was observed in 84.7% (*n* = 22) for OD and 96.3% (*n* = 25) for OS, with an overall mean response of 90.5% for both eyes (OPL). Using the gradient method, the calculated AC/A ratio had a mean value of 1.16 (SD = 1.05; range: 0–3.3), which was statistically significant (*p* = 0.003). An AC/A ratio of ≤3 was observed in 92.4% (*n* = 24) of patients, with 61.6% (*n* = 16) having an AC/A ratio of ≤1.

### 4.7. Analysis of the Relationship Between Selected Visual Parameters and Age

In the linear regression model, cases of non-convergence were replaced with a fixed NPC value of 40 cm. The analysis demonstrated a weak and statistically non-significant relationship between NPC and participants’ age (*p* > 0.05). The ANOVA yielded an F-value of 1.25 and *p* > 0.05, confirming the absence of significant differences between the age groups.

The AC/A ratio exhibited a decreasing trend with age. The highest values were observed among the youngest participants (4–5 years, M = 3.3), whereas the lowest values were recorded in the oldest group (14–16 years, M = 0.0). Statistical analysis indicated that these differences were significant (*p* < 0.01) and ANOVA confirmed a significant relationship between age and the AC/A ratio (F = 7.45, *p* < 0.01).

The results of dynamic retinoscopy (OD and OS) also showed a marked decline with age. Younger children (4–5 years) exhibited higher mean values (range: 3.0–4.0), whereas older participants (14–16 years) demonstrated significantly lower values (M = 1.5–1.6), which was statistically significant (*p* < 0.001). The ANOVA results further confirmed this relationship for both the right eye (OD: F = 10.23, *p* < 0.001) and the left eye (OS: F = 8.15, *p* < 0.001; Table 3).

**Figure 2:**
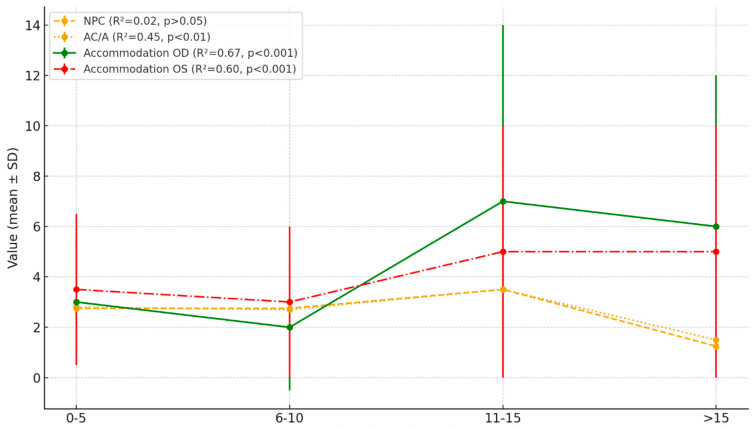
Visual parameters by age group in the STXBP1 cohort.

To provide a clearer overview of these trends, Figure 2 illustrates the mean values and standard deviations for each visual parameter across age groups.

The figure shows mean values with standard deviations (error bars) for near point of convergence (NPC, dashed line), AC/A ratio (dotted line), accommodative response in the right eye (solid line), and accommodative response in the left eye (dash–dot line) across four age groups (0–5, 6–10, 11–15, and >15 years). Regression analysis revealed no significant age-related effect for NPC (R^2^ = 0.02, *p* > 0.05), whereas the AC/A ratio significantly decreased with age (R^2^ = 0.45, *p* < 0.01). Both right and left eye accommodative responses showed a significant decline with age (OD: R^2^ = 0.67, *p* < 0.001; OS: R^2^ = 0.60, *p* < 0.001). These graphical data complement the statistical analysis presented in Table 3.

The relationship between age and refractive error was analyzed, focusing on hyperopia and astigmatism. Pearson’s test showed no significant correlation between age and hyperopia (r = −0.179; *p* = 0.240) or astigmatism (r = −0.053; *p* = 0.717). Spearman’s rank correlation confirmed the absence of linear associations (*p* = 0.505 for hyperopia; *p* = 0.717 for astigmatism).

**Figure 3:**
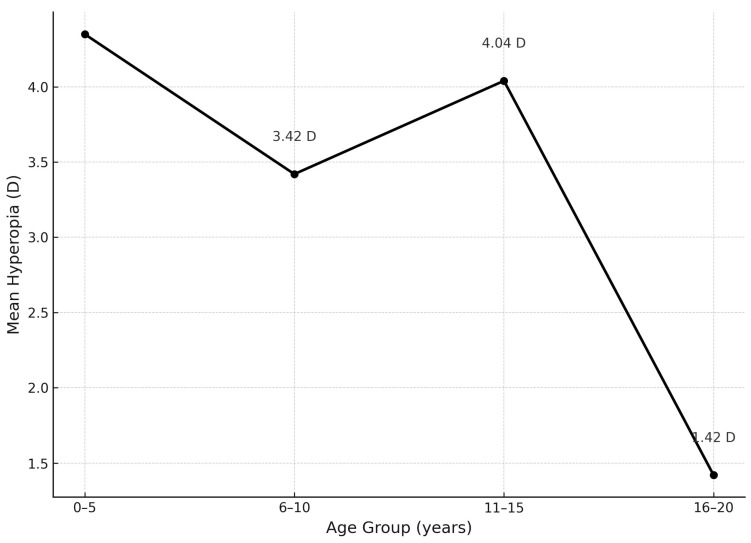
Change in mean hyperopia in relation to age of participants.

A comparison of the refractive error across four age groups (Group I: 0–5; Group II: 6–10; Group III: 11–15; Group IV: 16–20 years) using one-way ANOVA showed significant differences in hyperopia (F = 10.70; *p* < 0.0001). Mean hyperopia values were Group I: 4.35 D, Group II: 3.42 D, Group III: 4.04 D, and Group IV: 1.42 D. The most notable decline occurred in Group IV (*p* = 0.038). Although linear regression indicated a decrease of 0.11 D per year, the result was not statistically significant (*p* = 0.240), suggesting a non-linear course (Figure 3).

**Figure 4:**
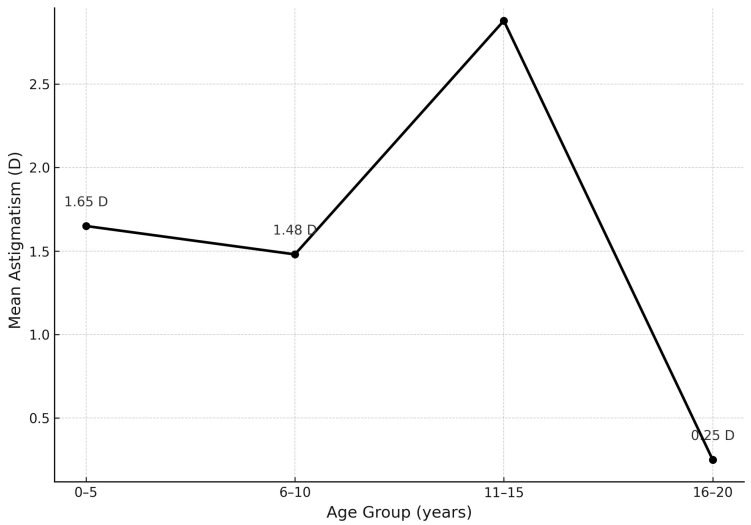
Change in mean astigmatism in relation to age of participants.

For astigmatism, ANOVA did not show significant differences (F = 2.71; *p* = 0.0079), though mean values varied: Group I: 1.65 D, Group II: 1.48 D, Group III: 2.88 D, and Group IV: 0.25 D. The Kruskal–Wallis test confirmed significant intergroup differences (H = 9.11; *p* = 0.028), especially in Group III, indicating a stepwise rather than linear pattern (Figure 4).

### 4.8. Mobility Status and Accommodative Parameters

In the analyzed cohort, 11 patients (42.3%) were able to walk independently, 4 patients (15.4%) required support during ambulation, and 8 patients (30.8%) were wheelchair-dependent. Comparative analysis of visual parameters across these three groups did not reveal statistically significant differences.

For accommodative response in the right eye, the mean values were 2.79 D in children walking independently, 2.38 D in children requiring assisted ambulation, and 2.47 D in wheelchair-dependent children. These differences were not statistically significant (ANOVA: F = 0.25, *p* = 0.78; Kruskal–Wallis: H = 0.12, *p* = 0.94).

For accommodative response in the left eye, the mean values were 2.89 D in children walking independently, 2.25 D in children requiring assisted ambulation, and 2.53 D in wheelchair-dependent children. No significant group effect was observed (ANOVA: F = 0.43, *p* = 0.66; Kruskal–Wallis: H = 0.34, *p* = 0.84).

For AC/A ratio, the mean values were 1.11 in children walking independently, 0.82 in children requiring assisted ambulation, and 1.42 in wheelchair-dependent children. Again, no statistically significant differences were found (ANOVA: F = 0.45, *p* = 0.64; Kruskal–Wallis: H = 0.18, *p* = 0.92).

### 4.9. Antiepileptic Treatment and Visual Parameters

In the studied cohort, the majority of patients (70%) received polytherapy (≥2 antiepileptic drugs), whereas only 30% were on monotherapy. The most frequently used drugs were vigabatrin in 6 children, valproate in 7 children, levetiracetam in 6 children, and carbamazepine in 2 children. The most common combination regimens included therapy with vigabatrin and valproate as well as therapy with levetiracetam and brivaracetam. A few patients also received lamotrigine, gabapentin, or rufinamide.

Comparative analysis showed that the accommodative response was most reduced in patients treated with regimens including vigabatrin and/or valproate (2.97 ± 1.23 D) compared with those receiving levetiracetam (2.50 ± 0.59 D) and carbamazepine (2.38 ± 1.24 D). Fusion was present in 66.7% of the vigabatrin/valproate group, 50% of the levetiracetam and carbamazepine groups, and 100% in the small and heterogeneous “other” group. The AC/A ratio was highest in the carbamazepine group (2.35 ± 0.49) and reduced in the vigabatrin/valproate (1.26 ± 1.25) and levetiracetam (0.90 ± 1.01) groups.

ANOVA didn’t show statistically significant differences in accommodative response between drug groups (*p* = 0.77). These results should be interpreted with caution, since most patients received combination therapy, making it difficult to attribute the observed deficits to a single drug.

## 5. Discussion

This is the first study to systematically evaluate visual function in children with pathogenic variants in the *STXBP1* gene, incorporating comprehensive ophthalmological and orthoptic assessments. The primary objective of the study was to characterize the visual phenotype and identify specific functional impairments in this pediatric population.

In the majority of cases, pregnancies were uneventful (88.5%), and most neonates achieved a maximum Apgar score, indicating the absence of significant perinatal risk factors. Nevertheless, profound developmental delays—motor (96.2%) and cognitive (84.7%)—as well as generalized hypotonia (88.6%) were observed, consistent with previously reported phenotypes associated with *STXBP1* epileptic encephalopathies [7,8]. Seizures occurred in 92.4% of patients during the first year of life, supporting prior epidemiological data reporting a prevalence of up to 95% [4,5,7,8].

Comprehensive ophthalmological examinations revealed no structural abnormalities in the anterior or posterior segment of the eye, and no pathology was observed in the ocular adnexa. Optical coherence tomography demonstrated preserved retinal architecture, with normal foveal and macular morphology. These findings are in line with earlier reports [13], suggesting that despite high retinal expression of *STXBP1* orthologs and syntaxin 3, the gene does not appear to play a critical role in retinal morphogenesis.

Nonetheless, functional impairments of photoreceptors and disrupted visual signal transmission remain plausible. This highlights the need for further diagnostic work-up, particularly electrophysiological testing of both the retina and visual pathways, to assess the full impact of *STXBP1* mutations on visual processing.

Although *STXBP1* is known to be expressed in the retina, there is currently no robust evidence linking its pathogenic variants with specific fundus abnormalities. In contrast, mutations in related genes such as *SSBP1* have been associated with optic atrophy and foveopathy, both of which present with clinically visible fundus changes [14]. Given that *STXBP1* encodes a protein critical for synaptic transmission, clinical parallels may exist with retinal dystrophies (e.g., *CACNA1F* mutations) or congenital stationary night blindness [15]. A 2007 study by Specht et al. [16] using a mouse model of photoreceptor synaptopathy demonstrated persistent structural and functional retinal alterations, further supporting this hypothesis.

Manifest latent nystagmus was identified in only one subject. Therefore, the previously proposed link between *STXBP1* and congenital nystagmus [11] remains unsubstantiated in this cohort, possibly due to the low prevalence of the condition in the study group.

Objective ocular cyclorotation (DFA) values were comparable with normative data reported in the literature. Findings by Shin et al. [17], Lengwiler et al. [18], and Simiera et al. [19] are consistent with those obtained in our cohort, confirming the absence of significant rotational anomalies in this patient population.

Refractive errors were highly prevalent, with hyperopia diagnosed in 76.9% of participants and astigmatism in 96.2%, substantially exceeding prevalence rates reported for the general population and for children with other developmental disorders [20,21].

Age-related refractive changes were also observed. Hyperopia showed a statistically significant reduction across age groups (ANOVA: F = 10.70, *p* < 0.0001), with the greatest decline occurring in the 16–20-year group, suggesting a delayed but ongoing emmetropization process. Similar trends were documented by Zhao et al. in a Chinese pediatric cohort [22], whereas emmetropization in the Polish population is reported to occur earlier, typically by age 7–8 [23].

Astigmatism also varied significantly across age groups (Kruskal–Wallis test, *p* = 0.028), with the most pronounced variation in the 11–15-year group. The lack of linear correlation with age, confirmed by Pearson and Spearman tests, supports earlier findings by Huynh et al. [24] and Czepita et al. [25] regarding the instability of astigmatic development during childhood.

Hypoaccommodation was identified in 90.5% of children, while 92.4% had a reduced AC/A ratio (≤3), including 61.6% with a ratio ≤ 1. These findings are in line with previous research demonstrating a high prevalence of accommodative dysfunction in neurodevelopmental disorders [26,27].

The analysis didn’t reveal any association between the level of motor function and accommodative–convergence parameters. Mean values of accommodative response and AC/A ratio were comparable among children who walked independently, required support, or were wheelchair-dependent. The absence of statistically significant differences suggests that, in the studied STXBP1 cohort, visual dysfunctions are not directly determined by motor limitations but rather arise from central mechanisms.

The elevated rate of hyperopia observed in this cohort may be attributable to delayed axial elongation of the eyeball. A plausible contributing factor is reduced visual stimulation due to cognitive and motor impairment. Children with neurodevelopmental delays often engage less in visual exploration, thereby reducing accommodative demand. Since accommodation is a physiological stimulus for axial eye growth and emmetropization, impaired accommodation may contribute both causally and consequentially to the persistence of hyperopia.

Convergence insufficiency, which was observed in over 50% of the cohort, may further compromise fixation stability and sustained visual attention. In our study, 61.6% of caregivers reported difficulties with eye contact and face fixation during infancy, an observation of clinical relevance given the behavioral overlap between *STXBP1* epileptic encephalopathy and autism spectrum disorder [7,8,28].

Similar visual deficits have been reported in other synaptopathies, including *DLG4* and *SYNGAP1*, in which oculomotor dysfunction, strabismus, and poor visual attention are frequently observed [9]. This supports the hypothesis that synaptic dysfunction affects not only higher-order cognitive processes but also visual sensory-motor integration.

Despite prior ophthalmological consultations in 73.2% of patients, only 34.7% were using corrective eyewear at the time of evaluation. Based on our findings, spectacle correction was recommended for 77% of the cohort. These results highlight a significant gap in vision care within this population. Early identification and correction of refractive errors are widely recognized as essential interventions to support optimal developmental outcomes in children with neurodevelopmental disorders [29,30].

In the present study, most patients were treated with polytherapy, reflecting the drug-resistant nature of STXBP1-related epileptic encephalopathy. This complicates the interpretation of individual drug effects, as visual function deficits may stem from both single-agent action and cumulative drug interactions. Notably, vigabatrin has been repeatedly linked to concentric visual field constriction and retinal nerve fiber layer (RNFL) thinning in both children and adults [31,32]. Valproate has also been associated with RNFL reduction in patients with epilepsy [33].

In our cohort, children on regimens including vigabatrin and/or valproate exhibited the most reduced accommodative response and lower AC/A values, while those treated with levetiracetam or carbamazepine showed relatively better visual outcomes. However, given the predominance of combination therapy, these findings should be interpreted with caution, serving as indications of possible trends rather than definitive evidence of a drug-specific effect. Further studies in larger, monotherapy-treated cohorts are needed to clarify these associations.

Epileptic encephalopathies such as Ohtahara syndrome, West syndrome, and Lennox–Gastaut syndrome are electroclinical diagnoses in which, in addition to very heterogeneous etiology, the factors determining the diagnosis are the semiology of the epileptic seizure, the patient’s age, neurodevelopmental condition, and pathological EEG graphoelements. Patients with a pathogenic variant of the STXBP1 gene may present with the phenotype of the above-mentioned electroclinical syndromes, and phenotypic evolution from one syndrome to another has also been observed. It should also be emphasized that not all patients are presenting the phenotypic evolution of specific phenotypic syndromes. In clinical practice, the diagnosis of a specific epilepsy syndrome has primarily a practical therapeutic dimension, while in the era of personalized medicine, we are increasingly aware of the molecular cause of electroclinical syndromes. Due to molecular heterogeneity, phenotypic diversity is also evident in the population of patients with STXBP1-related encephalopathy. For these reasons, it is appropriate to analyze a narrow population with a single molecular pathology, which may allow for the individualization of therapeutic strategies or the prevention of phenotypic progression in the future [4,34,35].

In the literature on other synaptopathies, such as SYNGAP1 and DLG4, visual dysfunctions are mainly described in terms of perceptual deficits and clinical ocular features (e.g., visual attention deficits, strabismus, hyperopia, nystagmus, and cases of cortical visual impairment) [36,37]. However, systematic quantitative analyses of orthoptic parameters are lacking. Against this background, our study represents the first comprehensive evaluation of quantitative visual functions in presynaptic synaptopathy due to STXBP1. It is distinguished by an exceptionally high prevalence of astigmatism (96.2%), predominant hypoaccommodation (>90%), a low AC/A ratio (≤1 in 61.6% of patients), and delayed emmetropization, accompanied by rare strabismus (7.7%) and absence of structural ocular pathology. These findings support the delineation of a distinct ophthalmic phenotype in STXBP1-related encephalopathy.

## 6. Limitations

This study is limited by its small sample size and lack of a control group, which restrict the generalizability and comparative interpretation of the findings. Electrophysiological and neuroimaging assessments were not performed, preventing evaluation of central visual pathway involvement. Additionally, the cross-sectional design precludes conclusions about the progression of visual function over time.

## 7. Conclusions

Refractive errors (particularly hyperopia and astigmatism), hypoaccommodation, and convergence insufficiency are commonly observed in children with *STXBP1*-related synaptopathy. These disturbances may significantly affect psychomotor and social development, including the ability for visual exploration and social interaction.

Importantly, most of these visual deficits are treatable. Early ophthalmological diagnostics, including cycloplegic refraction and orthoptic assessment, should be a standard element of care in children with synaptopathies. In cases where functional abnormalities are present despite normal ocular anatomy, implementing electrophysiological testing of the retina and visual pathways is also recommended.

## Figures and Tables

**Table 1:** Distribution of Hyperopia and Myopia After Cycloplegia in the studied group.

Refractive Error (D)	Hyperopia OD (%)	Hyperopia OS (%)	Hyperopia Mean OD/OS (%)	Myopia OD (%)	Myopia OS (%)	Myopia Mean OD/OS (%)
0.0 to 2.50	26.9% (*n* = 7)	38.5% (*n* = 10)	32.7%	3.8% (*n* = 1)	3.8% (*n* = 1)	3.8%
2.75 to 4.0	19.2% (*n* = 5)	34.6% (*n* = 9)	26.9%	0% (*n* = 0)	3.8% (*n* = 1)	1.9%
Above 4.25	15.4% (*n* = 4)	19.2% (*n* = 5)	17.3%	3.8% (*n* = 1)	0% (*n* = 0)	1.9%
Total	61.5% (*n* = 16)	92.3% (*n* = 24)	76.9%	7.7% (*n* = 2)	7.7% (*n* = 2)	7.7%

OD = right eye; OS = left eye.

**Table 2:** The distribution of astigmatism after cycloplegia in the studied group.

Refractive Error (D)	Astigmatism OD (%)/*n*	Astigmatism OS (%)/*n*	Astigmatism Mean OD/OS (%)
0.0 to 1.00	42.3% (*n* = 11)	53.8% (*n* = 14)	48%
1.25 to 2.5	30.8% (*n* = 8)	11.5% (*n* = 3)	21.2%
Above 2.75	23.1% (*n* = 6)	30.8% (*n* = 8)	27%
Total	96.2% (*n* = 25)	96.2% (*n* = 25)	96.2%

OD = right eye; OS = left eye.

**Table 3:** Statistical Analysis of Visual Parameters by Age Group.

Parameter	Mean (SD)	Regression Trend	R^2^	F	P
NPC	21.43 (15.3)	0.15	0.02	1.25	>0.05
AC/A	1.16 (1.05)	−0.15	0.45	7.45	<0.01
Dynamic Retinoscopy OD	2.63 (1.24)	−0.13	0.67	10.23	<0.001
Dynamic Retinoscopy OS	2.68 (1.31)	−0.12	0.60	8.15	<0.001

NPC = Near Point of Convergence; AC/A = Accommodative Convergence/Accommodation Ratio; OD = right eye; OS = left eye.

## Data Availability

Additional data are available from the corresponding author upon request.
